# Sex modifies the association between HIV and coronary artery disease among older adults in Uganda

**DOI:** 10.1002/jia2.25868

**Published:** 2022-01-07

**Authors:** Chris T. Longenecker, Milana Bogorodskaya, Seunghee Margevicius, Rashidah Nazzinda, Marcio Sommer Bittencourt, Geoffrey Erem, Sophie Nalukwago, Moises A. Huaman, Brian B. Ghoshhajra, Mark J. Siedner, Steven M. Juchnowski, David A. Zidar, Grace A. McComsey, Cissy Kityo

**Affiliations:** ^1^ University Hospitals of Cleveland Cleveland Ohio USA; ^2^ Case Western Reserve University Cleveland Ohio USA; ^3^ MetroHealth Medical Center Cleveland Ohio USA; ^4^ Joint Clinical Research Centre Kampala Uganda; ^5^ University of Sao Paulo Sao Paulo Brazil; ^6^ St. Francis Hospital Nsambya Kampala Uganda; ^7^ Makerere University School of Medicine Kampala Uganda; ^8^ University of Cincinnati College of Medicine Cincinnati Ohio USA; ^9^ Massachusetts General Hospital Boston Massachusetts USA; ^10^ Louis Stokes Cleveland Veterans Affairs Medical Center Cleveland Ohio USA

**Keywords:** HIV, sex, Uganda, cardiovascular diseases, monocytes, computed tomography angiography

## Abstract

**Introduction:**

Little is known about the epidemiology of coronary artery disease (CAD) in sub‐Saharan Africa, where the majority of people living with HIV (PLHIV) live. We assessed the association of HIV with CAD and explored relationships with monocyte activation in sex‐stratified analyses of older PLHIV and people without HIV (PWOH) in Uganda.

**Methods:**

The Ugandan Study of HIV effects on the Myocardium and Atherosclerosis (mUTIMA) follows 100 PLHIV on antiretroviral therapy (ART) and 100 age‐ and sex‐matched PWOH controls in Kampala, Uganda; all >45 years of age with >1 cardiovascular disease risk factor. At the year 2 exam (2017–2019), 189 participants had available coronary calcium score and 165 had coronary CT angiography (CCTA) for this analysis. A subset of participants (*n* = 107) had both CCTA and fresh whole blood flow cytometry for monocyte phenotyping.

**Results:**

Median age was 57.8 years and 63% were females. Overall, 88% had hypertension, 37% had diabetes and 4% were smokers. Atherosclerotic cardiovascular disease (ASCVD) risk was modestly higher for PWOH, but not statistically significant (median 10‐year ASCVD risk 7.2% for PLHIV vs. 8.6% for PWOH, *p* = 0.09). Median duration of ART was 12.7 years and 86% had suppressed viral load. Despite a high prevalence of risk factors, only 34/165 (21%, 95% CI 15–28%) had any coronary plaque. After adjustment for ASCVD risk score, HIV status was not associated with CAD (OR 0.55, 95% CI 0.23–1.30) but was associated with more severe CAD (segment severity score>3) among those with disease (OR 10.9, 95% CI 1.67–70.45). Females had a trend towards higher odds of CAD among PLHIV (OR 4.1, 95% CI 0.4–44.9), but a trend towards lower odds of CAD among PWOH (OR 0.30; 95% CI 0.07–1.3; HIV*sex interaction *p* = 0.019). CAD was positively correlated with classical monocytes (*r* = 0.3, *p* = 0.012) and negatively correlated with CX3CR1 expression (*r* = –0.31, *p* = 0.011) in PLHIV and negatively correlated with patrolling monocytes (*r* = –0.36, *p* = 0.031) and tissue factor expression (*r* = –0.39, *p* = 0.017) in PWOH.

**Conclusions:**

Our results suggest that HIV may be associated more with severity rather than the presence of CAD in Uganda. Sex differences in the HIV effect suggest that tailored CAD prevention strategies may be required in this setting.

## 1 INTRODUCTION

There are currently over 6 million persons living with HIV (PLHIV) aged 50 years and older, over half of whom live in sub‐Saharan Africa (SSA) [1]. Additionally, PLHIV are at higher risk of cardiovascular disease (CVD) compared to persons without HIV (PWOH) [2, 3]. Consequently, the global burden of HIV‐associated CVD has tripled in the last 20 years and is predicted to keep rising [2]. The mechanisms of HIV‐associated CVD include higher prevalence of traditional risk factors, HIV‐related factors and ongoing chronic immune dysregulation [3]. Innate immune mechanisms may be particularly important, as certain subsets of pro‐inflammatory monocytes and soluble markers of monocyte activation have been linked to coronary disease in multiple cohorts [4], including in a mechanistic sub‐study of the REPREIVE trial [5]. However, studies have been largely limited to males in high‐income countries (HICs), despite evidence that chronic HIV‐related immune activation is greater among females [6]. Little is known about the prevalence, sex differences and other risk factors for coronary artery disease (CAD) in SSA, where the majority of PLHIV live and where over 60% of PLHIV are females [7]. Therefore, we assessed the association of HIV with CAD prevalence and severity in sex‐stratified analyses and explored the relationship of CAD prevalence with markers of monocyte activation in persons living with and without HIV in Uganda.

## 2 METHODS

### 2.1 Study participants

The **U** gandan s **T** udy of H **I** V effects on the **M** yocardium and **A** therosclerosis (mUTIMA) is an ongoing prospective cohort study of PLHIV who are 1:1 matched with PWOH controls in Kampala, Uganda. PLHIV are recruited from the Joint Clinical Research Centre in Kampala. Age‐ (+/–3 years) and sex‐matched PWOH are recruited from internal medicine clinics in Kampala. On entry into the cohort, all PLHIV must be on antiretroviral therapy (ART) for >6 months with no changes in regimen within 12 weeks of enrolment. All participants, regardless of HIV status, must be older than 45 years with at least one CVD risk factor [hypertension, low high‐density lipoprotein cholesterol (HDL; <40 mg/dl for males or <50 mg/dl for females), diabetes mellitus, smoking or family history of early CAD]. Participants with a history of known CAD, peripheral artery disease, ischemic stroke, uncontrolled chronic inflammatory conditions, pregnancy, use of chemotherapy or immunomodulating agents, or an estimated glomerular filtration rate (eGFR) less than 30 ml/minute are excluded from entry into the cohort due to their potential confounding effect on measures of inflammation and immune activation. The protocol is approved by the University Hospitals Cleveland Medical Center Institutional Review Board, the Joint Clinical Research Centre Research Ethics Committee and the Uganda National Council for Science and Technology. All participants sign written informed consent.

One‐hundred PLHIV and 100 PWOH participants were enrolled into the original cohort from April 2015 to May 2017. Findings and methods from the baseline exam of the original cohort have been published previously [8, 9, 10]. From 2017 to 2019, participants were asked to return for a year 2 follow‐up exam. Those lost‐to‐follow‐up were replaced by age‐ and sex‐matched individuals to maintain a total cohort size of 200 participants. For this analysis, we included all participants with any cardiac CT data available. An additional subset had both cardiac CT and monocyte data. Monocytes were not available for all participants due to malfunctioning of the flow cytometer during the final months of the study period.

### 2.2 Clinical parameters

The year 2 exam consisted of two visits separated by 1 week. At the initial visit, clinical history was obtained from the medical record and confirmed using standardized questionnaires. Anthropometrics and blood pressure were measured by trained study staff. HIV status of control participants was confirmed with a rapid HIV test (HIV 1/2 STAT‐PAK®; Chembio, NY, USA; sensitivity 99.7% and specificity 99.9%). Blood was drawn after a 12‐hour fast for clinical labs performed at the JCRC, including a creatinine and eGFR, to determine the eligibility for CCTA. Ten‐year risk of atherosclerotic CVD was calculated using the pooled‐cohort equations for “other” race [11]. For PLHIV participants, the HIV viral load, nadir CD4 count and ART history were abstracted from the medical record.

### 2.3 Cardiac CT

**Figure 1:**
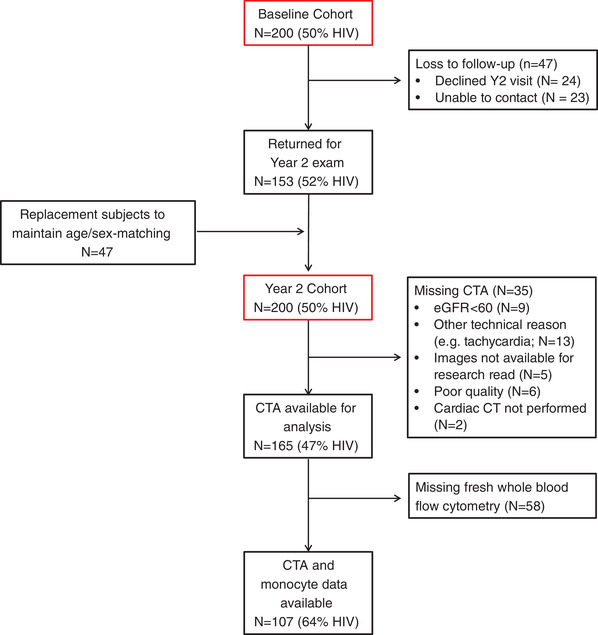
Derivation of the study population from the mUTIMA cohort. Abbreviations: CTA, computed tomography angiography; eGFR, estimated glomerular filtration rate.

After determining eligibility at the initial visit, participants returned to undergo CCTA on a 128‐slice Siemens Somatom scanner at Nsambya St. Francis Hospital in Kampala. Participants with eGFR >60 were eligible to have intravenous contrast for CCTA, although data acquisition for some participants was limited by other technical reasons, including inability to adequately lower the heart rate with beta‐blockers (Figure 1). The acquisition and image analysis protocols were developed in accordance with Society of Cardiovascular Computed Tomography Guidelines [12]. Two hours prior to the scan, participants were given 100 mg oral metoprolol with another 50 mg dose 30 minutes prior to the scan if the heart rate remained >60 beats per minute.

CT scans were read by a local radiologist for clinically significant findings and were then read offline in batch by a single expert reader (MSB) for research. The number of evaluable segments was determined per participant, and scans of poor technical quality were excluded from analysis. Using an 18‐segment model, there were some segments that were absent or not evaluable due to artefact. Because the average number of non‐evaluable segments per participant was modest and because of the low prevalence of disease in the study population, unevaluable segments were assumed to be normal for this analysis. Segment involvement score (SIS) was defined as the total number of diseased segments. Segment severity score (SSS) was calculated using a luminal obstruction weight for that segment (x1 if <25% obstruction, x2 if 25–50%, x3 if 50–70%, x4 if 70–99% and x5 if totally occluded), giving a maximum possible SSS of 90 for an 18‐segment model. Presence of CAD was defined as SIS>0 and more severe CAD was defined as an SSS greater than the median (i.e. SSS>3). High‐risk plaques were defined as plaques showing any of the following features: positive remodelling, spotty calcifications or napkin‐ring sign.

### 2.4 Monocyte subsets

Fresh whole blood flow cytometry was performed at the initial visit in real time using a MACSQuant® flow cytometer as previously described [13]. Samples were stained for the following markers: CD14, CD16, CD62p, CD69, CX3CR1, HLA‐DR and tissue factor (TF). Data were analysed by MAH and SMJ using FlowJo v10.

### 2.5 Statistical analysis

Participant baseline characteristics, laboratory values and CT variables were summarized as frequency (percentage) for categorical variables and median (IQR) for continuous variables. Baseline characteristics, presence of CAD, plaque distribution, CAC, SIS and SSS scores were compared by HIV status using the two‐sample *t*‐test or Wilcoxon rank sum test for continuous variables and Chi‐square or Fisher's exact tests for categorical variables, as appropriate.

For the primary analysis, logistic regression was used to assess the association of HIV status with presence of CAD (SIS>0), after adjustment for age, sex and ASCVD risk score. Since age and sex are components of the ASCVD risk score, variance inflation factors were checked to ensure that there was no multi‐collinearity. Then, an HIV*sex interaction term was added to the model to test for effect modification. Since the HIV*sex interaction was statistically significant, the effect of sex was modelled separately for PLHIV and PWOH and the effect of HIV was modelled separately for females and males. These stratified models were also adjusted for age and ASCVD score. A bar graph was created to visually depict plaque prevalence by HIV status and sex. Among PLHIV only, associations of HIV‐specific factors with presence of CAD (SIS >0) were explored using unadjusted logistic regression.

Similarly, the association of HIV status with more severe CAD (SSS>3) was assessed in logistic regression models adjusted for ASCVD risk. The HIV*sex interaction was not examined in this model due to small sample size and limited power.

Finally, in a pre‐specified analysis designed to limit the number of statistical tests performed, biserial correlation coefficients were used to assess the correlation between monocyte markers and presence of CAD separately by HIV status. Statistical significance was defined as *p*<0.05. All statistical analyses were performed using SAS v.9.4 (SAS Institute).

## 3 RESULTS

### 3.1 Study cohort

For the year 2 follow‐up exam, 153 of the original mUTIMA study participants returned and 47 were lost‐to‐follow‐up (24 declined and 23 were unable to be contacted; Figure 1). Those lost‐to‐follow‐up were replaced by age‐ and sex‐matched individuals to maintain a total cohort size of 200 participants. For this analysis, we included 189 participants (49% PLHIV) with available coronary artery calcium (CAC) score and 165 (47% PLHIV) with coronary CT angiography (CCTA). The most common reasons for missing angiography were tachycardia and poor renal function. An additional 107 participants (64% PLHIV) had both CCTA and monocyte data.

### 3.2 Baseline characteristics of study participants

The median age of the overall cohort was 57.0 years and 63% were females (Table 1). Median (IQR) age was similar by sex [57 (53–62) for females vs. 57 (51–62) for males, *p* = 0.54]. PLHIV had a lower body mass index [median (IQR) 27 (23–31) vs. 30 (26–33), *p* = 0.002] and were less likely to take blood pressure medication (61.0% vs. 78.0%, *p* = 0.009). There was a non‐significant trend towards PLHIV having less diabetes mellitus (30.0% vs. 43.0%, *p* = 0.056), having a higher HDL (54.3 vs. 51.0, *p* = 0.054) and having a lower ASCVD risk score [median (IQR) 7.2 (4.0–11.8) vs. 8.6 (4.2–16.1), *p* = 0.09]. Overall, prevalence of hypertension was high (87.5%), while prevalence of active tobacco use (4.0%) and excessive alcohol use (9.1%) was relatively low across both groups. Statin use was low overall (6%) and similar between groups. Among PLHIV, median nadir CD4+ cell count was 146 cells/mm^3^, median duration of ART was 13 years and 86% were fully virally suppressed (<20 c/ml; lower limit of assay detection). A quarter were taking protease inhibitors, 7% integrase strand transfer inhibitors and 7% abacavir. Most recent CD4+ cell count was not available due to the Uganda national guidelines recommending against routinely monitoring CD4+ cell counts once patients are virally suppressed. Compared to participants who completed both the baseline and the year 2 exam (*n* = 153), participants who were lost‐to‐follow‐up (*n* = 47) had higher median body mass index (BMI) (30.1 vs. 28.4 kg/m^2^, *p* = 0.02), but all other Table 1 characteristics were similar (all *p*>0.1). Among participants with CCTA and monocyte data, baseline characteristics were similar to the overall year 2 cohort (Table S1).

**Table 1:** Baseline characteristics of participants in the year 2 exam of the mUTIMA cohort study of older adults in Uganda

		PLHIV		PWOH		Overall	
	*N*	Median (IQR)	*N*	Median (IQR)	*N*	Median (IQR)	*p*‐Value
Demographics							
Age (years)	100	56.5 (53, 62)	100	57.5 (52, 63)	200	57 (53, 62)	0.36
Sex (female)	100	63 (63%)	100	63 (63%)	200	126 (63%)	1.00
Greater than secondary education	100	31 (31%)	100	40 (40%)	200	71 (35.5%)	0.24
*Occupation*							
Farmer	100	21 (21%)	100	24 (24%	200	45 (22.5%)	0.12
Selling goods	100	13 (13%)	100	22 (22%)	200	35 (17.5%)	
Unemployed	100	24 (24%)	100	13 (13%)	200	37 (18.5%)	
Other^a^	100	42 (42%)	100	41 (41%)	200	83 (41.5%)	
Medical history							
Diabetes	100	30 (30%)	100	43 (43%)	200	73 (36.5%)	0.06
Hypertension	100	86 (86%)	100	89 (89%)	200	175 (87.5%)	0.52
Any prevalent CVD^b^	100	4 (4%)	100	4 (4%)	200	8 (4%)	1.00
*MI* ^b^	100	0	100	0	200	0	NA
*Stroke* ^b^	100	2 (2%)	100	0	200	2 (1%)	0.50
CVD risk factors							
Body mass index (kg/m^2^)	100	27 (23, 31)	100	30 (26, 33)	200	29 (25, 33)	0.002
Waist:hip ratio	100	0.91 (0.86, 0.95)	100	0.89 (0.84, 0.94)	200	0.90 (0.84, 0.94)	0.21
Systolic blood pressure (mmHg)	100	149 (130, 169)	100	147 (132, 169)	200	148 (132, 169)	0.91
Total cholesterol (mg/dl)	100	208 (177, 235)	100	193 (177, 228)	200	200 (177, 232)	0.38
LDL (mg/dl)	100	130 (107, 157)	99	130 (112, 163)	199	130 (108, 160)	0.33
HDL (mg/dl)	100	54 (44, 68)	100	51 (43, 61)	200	53 (43, 65)	0.05
eGFR_cr_ (ml/minute/1.73 m^2^)	100	104 (87, 155)	100	104 (90, 115)	200	104 (89, 115)	0.87
Any BP medication	100	61 (61%)	100	78 (78%)	200	139 (69.5%)	0.009
Statin	100	5 (5%)	100	7 (7%)	200	12 (6%)	0.55
Any alcohol	98	23 (23%)	96	29 (30%)	194	52 (27%)	0.30
Harmful alcohol^c^	16	2 (12.5%)	28	2 (7%)	44	4 (9%)	0.61
Current smoker	100	4 (4%)	100	4 (4%)	200	8 (4%)	1.00
10‐year ASCVD risk score (%)^d^	100	7.2 (4.0, 11.8)	100	8.6 (4.2, 16.1)	200	7.9 (4.1, 13.4)	0.09
HIV characteristics							
Nadir CD4+ count (cells/mm^3^)	86	146 (64, 261)		NA		NA	
HIV viral load suppressed	97	83 (86%)		NA		NA	
VL if not suppressed (copies/ml)	14	70 (50, 229)		NA		NA	
HIV duration (years)	99	13.9 (11.6, 15.2)		NA		NA	
ART duration (years)	99	12.7 (9.9, 14.1)		NA		NA	
Current protease inhibitor	100	25 (25%)		NA		NA	
Current integrase inhibitor	100	7 (7%)		NA		NA	
Current abacavir	100	7 (7%)		NA		NA	

Abbreviations: ART, antiretroviral therapy; ASCVD, atherosclerotic cardiovascular disease; BP, blood pressure; CVD, cardiovascular disease; eGFR, estimated glomerular filtration rate; HDL, high‐density lipoprotein; IQR, interquartile range; LDL, low‐density lipoprotein; MI, myocardial infarction; PLHIV, people living with HIV; PWOH, people without HIV; VL, viral load.

^a^ Other occupation includes teacher, military/police/security, trucker/driver/conducter, construction worker, healthcare worker, business person (other than selling goods), government/clerical/secretarial, mechanic and other/not‐listed (each <10% overall).

^b^ Although prevalent CVD is an exclusion criterion at study entry, some participants developed CVD during the initial 2 years of longitudinal follow‐up.

^c^ Harmful use assessed only among those with any alcohol use.

^d^ 10‐year ASCVD risk score calculated using the pooled cohort equations and “other” race term.

### 3.3 Low prevalence and severity of CAD in the overall population

For the CCTA analysis (*n* = 165), the quality of analysed scans was good overall, with a median (IQR) number of evaluable segments per participant of 14 (13–15), without difference by HIV status (*p* = 0.69). Despite a high prevalence of risk factors, a large majority of participants (81%) had no CAC or plaque (SIS = 0) and only 2% had CAC>300 (Table 2). The CCTA data confirmed that only 34/165 (21%, 95% CI 15–28%) had any coronary plaque and most disease was mild. Of the 34 participants with detectable CAD, the median (IQR) number of diseased segments was 2 (1, 3) with a median (IQR) SSS score of 3 (2, 6). Of *n* = 85 total plaques across both groups, the vast majority (85%) were calcified or partially calcified. There were no participants with high‐risk coronary plaque features.

**Table 2:** Measures of coronary artery disease severity among older adults living in Uganda, stratified by HIV status

Coronary artery calcium scoring		PLHIV	PWOH	Overall	*p*‐Value
CAC (*N* = 189)	0	79 (85.87%)	75 (77.32%)	154 (81.48%)	0.4886*
	1–100	9 (9.78%)	14 (14.43%)	23 (12.17%)	
	101–300	3 (3.26%)	5 (5.15%)	8 (4.23%)	
	>300	1 (1.09%)	3 (3.09%)	4 (2.12%)	
Coronary CT angiography					
Number of evaluable segments per person (*N* = 165)^a^	14.00 (12.00, 15.00)	14.00 (13.00, 15.00)	14.00 (13.00, 15.00)	0.6863**	
SIS score >0 (*N* = 165)	67 (85.90%)	64 (73.56%)	131 (79.39%)	0.0296*	
Median SIS score among *N* = 34 with SIS >0	3.00 (1.00, 6.00)	2.00 (1.00, 2.00)	2.00 (1.00, 3.00)	0.0697**	
Median SSS score among *N* = 34 with SIS >0	5.00 (2.00, 9.00)	2.00 (1.00, 3.00)	3.00 (2.00, 6.00)	0.0244**	

Note: All values displayed as median (interquartile range) or number (%).

Abbreviations: CAC, coronary artery calcium score; PLHIV, people living with HIV; PWOH, people without HIV; SIS, segment involvement score; SSS, segment severity score.

^a^ Total possible segments are 18, but some segments are missing or not evaluable due to artefact. For all subsequent analyses, unevaluable segments were assumed to be normal given the low prevalence of disease overall.

* *p*‐Values from Chi square/Fisher's Exact test.

** *p*‐Values from Wilcoxon rank sum test.

### 3.4 Presence and severity of CAD by HIV status and sex

**Figure 2:**
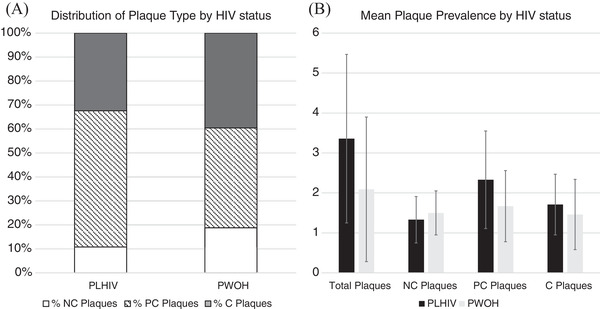
Distribution of coronary plaque among older adults in Uganda with any plaque (*n* = 34), stratified by HIV status. (a) Plaque subtype distribution by HIV status. The proportion of each plaque type did not differ between PLHIV and PWOH (all *p*>0.15); and (b) mean prevalence of coronary plaque by HIV status and plaque subtype. Error bars represent standard deviation. Abbreviations: C, calcified plaque; PC, partially calcified plaque; PLHIV, people living with HIV; PWOH, people without HIV; NC; non‐calcified plaque. All *p*>0.15 except for total plaques (*p* = 0.06).

**Figure 3:**
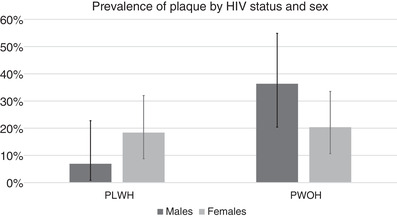
Prevalence of CAD among older males and females in Uganda with available coronary computed tomography angiography data (*n* = 165), stratified by HIV status. In a multivariable model adjusted for age, sex, ASCVD score and HIV status, an HIV*sex interaction was statistically significant (*p* = 0.0194). Solid bars show the point prevalence with error bars representing the 95% confidence interval. Abbreviations: ASCVD, atherosclerotic cardiovascular disease; PLHIV, people living with HIV; PWOH, people without HIV.

In unadjusted comparisons (Table 2), PLHIV had a modestly lower overall prevalence of detectable CAD (14% vs. 26%, *p* = 0.05); however, among those with CAD, median SSS (5.0 vs. 2.0, *p* = 0.02) and median SIS score were higher (3.0 vs. 2.0, *p* = 0.07) in PLHIV compared to PWOH. In adjusted logistic regression models, HIV status was not associated with prevalence of CAD (adj OR 0.55, 95% CI 0.23–1.30, *p* = 0.17; Table 3a) but was associated with more severe CAD (adj OR 10.9, 95% CI 1.7–70.4, *p* = 0.01; Table 3b) among those with CAD. Plaque‐type distribution did not differ between PLHIV and PWOH (Figure 2; all *p*>0.15), with the majority of plaques being partially calcified (PLHIV 57%; PWOH 42%) or calcified (PLHIV 32%; PWOH 40%) plaques. A prominent and statistically significant HIV*sex interaction existed for the fully adjusted model (*p* for interaction = 0.019; Figure 3). Although CIs were wide, females had a trend towards higher odds of CAD among PLHIV (OR 4.1, 95% CI 0.4–44.9), but had a trend towards lower odds of CAD among PWOH (OR 0.30; 95% CI 0.07–1.3) as shown in Table 4a. The effect of HIV is shown separately for females and males in Table 4b. Among PLHIV only, nadir CD4+, HIV‐1 viral load, HIV duration, ART duration, current protease inhibitor and current integrase inhibitor use were not associated with presence of CAD (all *p*>0.05).

**Table 3:** Multivariable adjusted models of the HIV effect on (a) presence of any coronary artery disease (segment involvement score >0) among *n* = 165 older adults in Uganda with available coronary computed tomography angiography data and (b) severity of disease (segment severity score >3) among the *n* = 34 patients with any coronary artery disease

(a). Presence of CAD (SIS>0; *n* = 165)	OR	95% CI	*p*‐Value
HIV status (+)	0.545	(0.228, 1.302)	0.1719
Age (per year)	1.121	(1.025, 1.227)	0.0125
Sex (female)	0.854	(0.286, 2.545)	0.7763
ASCVD risk (per 1% absolute increase in risk)	1.024	(0.967, 1.085)	0.4111

(b). Severity of CAD (SSS >3; *n* = 34)	OR	95% CI	*p*‐Value
HIV status (+)	10.925	(1.674, 70.446)	0.0119
ASCVD risk (per 1% absolute increase in risk)	1.046	(0.985, 1.110)	0.1440

Abbreviations: ASCVD, atherosclerotic cardiovascular disease; CI, confidence interval; OR, odds ratio; SIS, segment involvement score; SSS, segment severity score.

**Table 4:** (a) Association of sex with prevalent coronary artery disease among older adults in Uganda, stratified by HIV status, and (b) association of HIV status with prevalent coronary artery disease, stratified by sex

(a)		Adjusted model		
		Adj OR	95% CI	*p*‐value
PLHIV	*Sex (female)*	4.144	(0.383, 44.855)	0.2420
	*Age (per year)*	1.089	(0.948, 1.250)	0.2293
	*ASCVD risk (per 1%)*	1.028	(0.902, 1.171)	0.6776
PWOH	*Sex (female)*	0.298	(0.068, 1.305)	0.1081
	*Age (per year)*	1.172	(1.026, 1.338)	0.0190
	*ASCVD risk (per 1%)*	1.020	(0.948, 1.096)	0.5966

(b)		Adjusted model		
		Adj OR	95% CI	*p*‐value
Males	*HIV status (+)*	0.109	(0.019, 0.627)	0.0130
	*Age (per year)*	1.147	(0.972, 1.353)	0.1044
	*ASCVD risk (per 1%)*	1.002	(0.917, 1.095)	0.9604
Females	*HIV status (+)*	1.443	(0.465, 4.479)	0.5259
	*Age (per year)*	1.113	(0.994, 1.247)	0.0642
	*ASCVD risk (per 1%)*	1.065	(0.968, 1.172)	0.1958

Abbreviations: Adj OR, adjusted odds ratio; ASCVD, atherosclerotic cardiovascular disease; CI, confidence interval; PLHIV, people living with HIV; PWOH, people without HIV.

### 3.5 Correlation of CAD and monocyte subtypes and activation markers by HIV status

**Figure 4:**
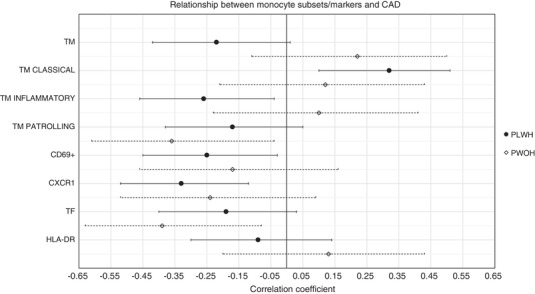
Correlation of total monocyte count, monocyte subsets and monocyte activation markers with presence of coronary artery disease among older adults in Uganda, stratified by HIV status. Point estimates represent the biserial correlation coefficient and error bars represent 95% confidence interval. Abbreviations: HLA, human leukocyte antigen; PLHIV, people living with HIV; PWOH, people without HIV; TM, total monocyte; TF, tissue factor.

Figure 4 shows the correlation of monocyte subsets with prevalent CAD stratified by HIV status. In PLHIV, CAD positively correlated with classical monocytes (*r* = 0.30, *p* = 0.01) and negatively correlated with inflammatory monocytes (*r* = –0.24, *p* = 0.05), CD69+ expression (*r* = –0.25, *p* = 0.04) and CX3CR1 expression (*r* = –0.31, *p* = 0.01). In PWOH, CAD negatively correlated with patrolling monocytes (*r* = –0.36, *p* = 0.03) and TF expression (*r* = –0.39, *p* = 0.02).

## 4 DISCUSSION

To our knowledge, this is the first study to examine HIV effects on coronary artery plaque in an SSA population. We demonstrate no association of HIV status with overall presence of coronary disease, but in our study, HIV was associated with severity of CAD among those with disease. These findings suggest that, in SSA, HIV status may have a stronger effect on disease progression (i.e. severity), rather than disease initiation (i.e. prevalence), although such findings need to be confirmed in longitudinal studies. Our results further suggest that after adjusting for ASCVD risk score, females with HIV may have higher risk of subclinical CAD compared to males, whereas females without HIV may have lower risk than males. The associations of disease with monocyte subsets were unexpected, and these findings require further investigation as discussed below.

There is an emerging literature of CCTA studies among PLHIV in HICs, with most showing some association of HIV status with disease [14, 15, 16, 17], although at least one prominent cohort study found no HIV effect in an initial cross‐sectional analysis [18] and subsequent longitudinal analysis [19]. Thus, our findings of an association between HIV and coronary disease severity in the SSA context are confirmatory of studies in HIC. Some studies from HIC have noted higher prevalence of non‐calcified plaque in PLHIV compared to PWOH. In contrast, non‐calcified plaque was less common and not associated with HIV in our study. Prevalence of non‐calcified plaque varies widely (20–60% among PLHIV and 5–52% among PWOH) [20], possibly due to differences in both participant characteristics and methodology. Studies that compare participants across geographic settings using the same protocols for CCTA acquisition and interpretation are needed.

The discrepancy between the HIV effect on overall prevalence of disease (SIS >0) and disease severity (SSS >3) in our study is interesting. Previous literature has demonstrated that different risk factors are associated with initial CAC scores and progression of CAC scores, suggesting different pathophysiology of CAD development and progression [21]. Whether similar differences exist for PLHIV is unknown. Larger and longer longitudinal cohorts should further examine this question among PLHIV in SSA.

The most important finding from our study may be that sex appears to modify the association of HIV with CAD. Whether this is driven primarily by factors that may lower CAD risk for males with HIV or increase risk for females (vs. a combination of both) is not clear. These trends persisted after adjustment for global ASCVD risk, suggesting that non‐traditional risk factors may be at play. Sex differences among PLHIV may differ by geographic location, race and cultural practices. For example, in Uganda, females with and without HIV are exposed to more carbon monoxide via indoor cook stoves compared to males [22], which is known to be associated with increased CVD mortality [23]. This may partly explain why inflammation markers are higher in Ugandan females compared to males [24]. Life course differences in lifestyle and preventive medical care may also differ by sex and HIV status. The older age distribution of females in our study means that the vast majority had likely completed menopause. Females with HIV are known to have premature ovarian failure, which may contribute to CVD risk [6]. Future studies should investigate the role of reproductive ageing on CVD risk—including CAD characteristics—among females in SSA.

Our findings clearly contrast with those of a U.S.‐based study of PLHIV only (no PWOH controls), in which females had nearly four‐fold lower odds of any coronary plaque compared to males [25]. In that study, females also had lower risk of high‐risk plaque features. No high‐risk plaque features were identified in our cohort, which had an overall low prevalence of generally mild disease. This is consistent with our prior report from the baseline exam of mUTIMA [8], in which over 90% of Ugandans had no detectable CAC. In that study, Ugandans had 14‐fold lower odds of detectable CAC compared to a U.S.‐based cohort after adjustment for traditional CVD risk factors. In this year 2 follow‐up CCTA study, the prevalence of CAC was slightly higher than the baseline study, but the prevalence of any CAD—including non‐calcified plaque—was still only 21%. The reasons for low prevalence of CAD are likely multifactorial, but may relate to more favourable life course exposure to risk factors or favourable lipids. Our mUTIMA cohort and other ongoing cohort studies in the SSA region should continue to investigate the epidemiology and potential mechanisms of coronary disease among PLHIV, including the role of sex.

The innate immune system may play an important role in HIV‐associated cardiovascular risk [26]. Circulating monocytes can be divided into three subsets based on CD14 and CD16 expression: “classical” (CD14^++^CD16^−^), “inflammatory” or “intermediate” (CD14^++^CD16^+^) and “patrolling” or “non‐classical” (CD14^low/+^CD16^++^) [27]. Previous studies have described more intermediate and non‐classical monocytes in patients with chronic conditions, such as obesity or stable CAD [28], including treated and untreated HIV infection [29]. Although many studies have linked soluble markers of monocyte activation, such as sCD14 and sCD163 with atherosclerosis in PLHIV [4], evidence for an association between monocyte subsets and CAD is less robust. Both intermediate monocytes [30] and non‐classical monocytes [31] have been associated with initial CAC scores as well as CAC score progression. In our study, we aimed to assess the associations of CAD with these three monocyte subsets and also further explore additional cell‐surface markers that have been postulated to play a role in HIV‐related atherosclerosis. For example, CX3CR1+ CD8+ T cells are present in high numbers in human atherosclerotic plaques and aortas of rhesus macaques with simian immunodeficiency virus (SIV) [32]. Monocyte TF expression is elevated in PLHIV, mirroring the monocyte profile of PWOH with acute coronary syndromes [33]. Finally, latent TB was associated with HLA‐DR expression on monocytes in our mUTIMA cohort [13] and with obstructive CAD in a combined analysis of our data and a similar cohort in Peru [34]. Therefore, our findings that CAD negatively correlated with inflammatory monocytes, CD69+ expression and CX3CR1 expression among PLHIV and negatively correlated with non‐classical monocytes and TF expression in PWOH were unexpected. Future studies with larger samples of people with prevalent disease should assess whether correlations between CAD and cell surface markers are modified by monocyte subset or clinical characteristics.

Our study was strengthened by the large proportion of females, a well‐matched PWOH control population, use of the state‐of‐the‐art CCTA methods to diagnose prevalence and severity of CAD disease and incorporation of flow cytometry to identify cellular markers of immune activation. Nonetheless, our study also has limitations. Although demonstrating a low prevalence of CAD in this risk‐factor enriched cohort is a major study finding, the low prevalence of disease limited statistical power to explore multivariable associations. Although our study was larger than the initial studies examining the effect of HIV [16, 35] and inflammation [36] on CT coronary plaque among males and females in the United States, we acknowledge that the sample size also limited some of our analyses. Although 23% of participants from our baseline study [8] did not return for the year 2 exam, the characteristics of these participants were similar to those who did contribute data to this study. Our analysis of HIV disease characteristics and ART was limited by sample size and lack of current CD4+ count. We did not find an association of ART categories with CAD, but cannot exclude an effect on clinical ASCVD events in SSA as has been shown in HIC [37, 38, 39]. Ultimately, as with all cross‐sectional studies, ours is not able to determine causal relationships among the variables studied. Finally, our study focused on older individuals with well‐controlled HIV and highly prevalent CVD risk factors, thus findings may not be applicable to younger, lower risk populations, those with more advanced kidney disease or those without HIV disease control.

## 5 CONCLUSIONS

CAD among PLHIV will continue rising as the population ages. However, the prevalence and characteristics of HIV‐related CAD in SSA may differ from HIC. This study demonstrates that despite a high prevalence of traditional risk factors, the burden of sub‐clinical CAD is low in this Ugandan cohort. Our findings suggest that HIV may be associated more with progression rather than initiation of CAD, and that sex may modify the HIV effect. Lastly, prior studies have associated innate immune activation with CAD among PLHIV; therefore, our surprising findings of an inverse correlation between CAD and some inflammatory monocyte subsets require further investigation.

## COMPETING INTERESTS

There are no competing interests relevant to the presented work. Outside of the current work, CTL has received research grants from Gilead Sciences and Medtronic Foundation and has served on an advisory board for Esperion Therapeutics. MSB has received a research grant from Sanofi, consulting fees from Bayer, and speaker fees from Novartis, NovoNordisk and GE Healthcare. GAM has served as a scientific advisor for Gilead Sciences, ViiV and Merck; has received research grants from Bristol‐Myers Squibb, Merck, Astellas, Tetraphase, Roche and Gilead Sciences. All other authors have no disclosures.

## AUTHORS’ CONTRIBUTIONS

CTL, MAH, BBG, MJS, DAZ, GAM and CK designed the study. CTL and MB drafted the manuscript. SM performed the statistical analyses. CTL, RN, MSB, GE, SN, MAH and SMJ performed the research. All authors critically reviewed and edited the manuscript for content.

## FUNDING

This work was supported by the National Heart, Lung, and Blood Institute of the National Institutes of Health (K23 HL123341 to CTL and R01 HL141053 to MJS).

## DISCLAIMER

The content is solely the responsibility of the authors and does not necessarily represent the official views of the National Institutes of Health.

## Supporting information


**Table S1**. Baseline characteristics of participants with monocyte activation markers and CTA data from Year 2 cohort

## Data Availability

The data that support the findings of this study are available from the corresponding author [CTL] upon reasonable request.
