# Supplementary material for: Sex modifies the association between HIV and coronary artery disease among older adults in Uganda
Source: J Int AIDS Soc. 2022 Jan 7;25(1):e25868. doi: 10.1002/jia2.25868 (PMC8741262; doi:10.1002/jia2.25868)
Supplement: Supplementary file 1 — Table S1. Baseline characteristics of participants with monocyte activation markers and CTA data from Year 2 cohort [file JIA2-25-e25868-s001.docx]

**Supplemental Table 1**. **Baseline characteristics of participants with monocyte activation markers and CTA data from Year 2 cohort**

|  |  | **PLWH** |  | **PWOH** |  | **Overall** |  |
| --- | --- | --- | --- | --- | --- | --- | --- |
|  | **N** | **Median (IQR)** | **N** | **Median (IQR)** | **N** | **Median (IQR)** | **p-value** |
| **Demographics** | | | | | | | |
| Age (years) | 69 | 55 (53, 60) | 38 | 62 (51, 66) | 107 | 57 (52.00, 63.00) | 0.06 |
| Sex (female) | 69 | 42 (61%) | 38 | 27 (71%) | 107 | 69 (64%) | 0.29 |
| **Medical History** | | | | | | | |
| Diabetes | 69 | 15 (22%) | 38 | 14 (37%) | 107 | 29 (27%) | 0.09 |
| Hypertension | 69 | 60 (87%) | 38 | 34 (89%) | 107 | 94 (88%) | 0.77 |
| Any prevalent CVD^*^ | 69 | 3 (4.4%) | 38 | 2 (5.3%) | 107 | 5 (4.7%) | 1.00 |
| *MI*^*^ | 69 | 0 | 38 | 0 | 107 | 0 | NA |
| *Stroke*^*^ | 69 | 2 (2.9%) | 38 | 0 | 107 | 2 (1.9%) | 0.54 |
| **CVD Risk Factors** | | | | | | | |
| Body Mass Index (kg/m^2^) | 69 | 27 (24, 31) | 38 | 31 (27, 34) | 107 | 29 (25, 33) | 0.01 |
| Waist:Hip Ratio | 69 | 0.91 (0.87, 0.95) | 38 | 0.90 (0.85, 0.95) | 107 | 0.91 (0.86, 0.95) | 0.38 |
| Systolic Blood Pressure (mmhg) | 69 | 149 (133, 174) | 38 | 142 (130, 165) | 107 | 147 (130, 171) | 0.19 |
| Total Cholesterol (mg/dL) | 69 | 208 (172, 238) | 38 | 192 (173, 213) | 107 | 201 (172, 235) | 0.48 |
| LDL (mg/dL) | 69 | 129 (108, 156) | 38 | 127 (108, 160) | 107 | 129 (108, 158) | 0.47 |
| HDL (mg/dL) | 69 | 55 (44, 71) | 38 | 47 (42, 63) | 107 | 54 (43, 68) | 0.09 |
| eGFR_cr_ (ml/min/1.73m^2^) | 69 | 109 (97, 117) | 38 | 107 (95, 115) | 107 | 108 (96, 116) | 0.68 |
| Taking BP Medication | 69 | 43 (62%) | 38 | 30 (79%) | 107 | 73 (68%) | 0.08 |
| Any alcohol | 68 | 14 (21%) | 35 | 11 (31%) | 103 | 25 (24%) | 0.22 |
| Harmful alcohol^‡^ | 8 | 2 (25%) | 9 | 0 | 17 | 2 (12%) | 0.21 |
| Current Smoker | 69 | 3 (4.4%) | 38 | 0 | 107 | 3 (2.8%) | 0.55 |
| 10-year ASCVD Risk Score (%)^†^ | 69 | 7.1 (4.1, 10.6) | 38 | 9.3 (3.5, 13.4) | 107 | 7.4 (3.6, 12.3) | 0.11 |
| **HIV Characteristics** | | | | | | | |
| Nadir CD4+ count (cells/mm^3^) | 62 | 162 (58, 264) |  | NA |  | NA |  |
| HIV viral load suppressed | 68 | 58 (85%) |  | NA |  | NA |  |
| VL if not suppressed (copies/ml) | 10 | 128 (51, 254) |  | NA |  | NA |  |
| HIV Duration (years) | 69 | 14.0 (12.0, 15.5) |  | NA |  | NA |  |
| ART Duration (years) | 69 | 12.7 (10.1, 14.1) |  | NA |  | NA |  |
| Current Protease Inhibitor | 64 | 19 (30%) |  | NA |  | NA |  |
| Current Integrase Inhibitor | 68 | 2 (2.9%) |  | NA |  | NA |  |

^*^ Although prevalent CVD is an exclusion criterion at study entry, some participants developed CVD during the initial 2 years of longitudinal follow-up. ^‡^ Harmful use assessed only among those with any alcohol use. ^†^10-year ASCVD risk score calculated using the pooled cohort equations and “other” race term. CVD, cardiovascular disease; MI, myocardial infarction; LDL, low density lipoprotein; HDL, high density lipoprotein; eGFR; estimated glomerular filtration rate; BP, blood pressure; ASCVD, atherosclerotic cardiovascular disease; VL, viral load; ART, antiretroviral therapy.
